# Triggering of Suicidal Erythrocyte Death by Penta-*O*-galloyl-β-d-glucose

**DOI:** 10.3390/toxins6010054

**Published:** 2013-12-24

**Authors:** Kousi Alzoubi, Sabina Honisch, Majed Abed, Florian Lang

**Affiliations:** Department of Physiology, University of Tuebingen, Gmelinstraße 5, 72076 Tuebingen, Germany; E-Mails: kossai.z@gmail.com (K.A.); honisch.s@gmx.de (S.H.); dr.magd81@hotmail.com (M.A.)

**Keywords:** cell membrane scrambling, phosphatidylserine, calcium, cell volume, eryptosis

## Abstract

The polyphenolic 1,2,3,4,6-penta-*O*-galloyl-beta-d-glucose from several medicinal herbs triggers apoptosis and has, thus, been proposed for treatment of malignancy. The substance is at least partially effective through caspase activation. In analogy to apoptosis of nucleated cells, erythrocytes may enter suicidal death or eryptosis, which is characterized by cell shrinkage and by phosphatidylserine translocation to the erythrocyte surface. Eryptosis is triggered by increase of cytosolic Ca^2+^-activity ([Ca^2+^]i). The sensitivity to [Ca^2+^]i is enhanced by ceramide. The present study explored whether penta-*O*-galloyl-β-d-glucose stimulates eryptosis. Cell volume was estimated from forward scatter, phosphatidylserine exposure from annexin V binding, hemolysis from hemoglobin-release, [Ca^2+^]i from Fluo3-fluorescence and ceramide abundance from fluorescent antibodies. A 48-h exposure of human erythrocytes to penta-*O*-galloyl-β-d-glucose significantly decreased forward scatter (50 µM) and significantly increased annexin V binding (10 µM). Up to 50 µM penta-*O*-galloyl-β-d-glucose did not significantly modify [Ca^2+^]i. However, the effect of penta-*O*-galloyl-β-d-glucose (25 µM) induced annexin V binding was slightly, but significantly, blunted by removal of extracellular Ca^2+^, pointing to sensitization of erythrocytes to the scrambling effect of Ca^2+^. Penta-*O*-galloyl-β-d-glucose (25 µM) further increased ceramide formation. In conclusion, penta-*O*-galloyl-β-d-glucose stimulates suicidal erythrocyte death or eryptosis, an effect partially due to stimulation of ceramide formation with subsequent sensitization of erythrocytes to Ca^2+^.

## 1. Introduction

1,2,3,4,6-Penta-*O*-galloyl-beta-d-glucose, a polyphenolic compound from medicinal herbs [1], has been proposed for the treatment of several disorders including cancer and diabetes [1]. Its activity against malignancy has been attributed to inhibition of angiogenesis [1,2], cell proliferation [1,3,4], DNA replication [1,2,3], inflammation [1,5], oxidative stress [1,6,7], as well as induction of cell cycle arrest [1,2,3,4,5,8] and apoptosis [1,2,3,8,9,10,11,12,13,3,8]. Signaling mediating the effects of penta-*O*-galloyl-beta-d-glucose include p53 [1,7], JAK-Stat [1,11], COX-2 [1,12], VEGFR1 [1,12], AP-1 [1], SP-1 [1], Nrf-2 [1], MMP-9 [1], jun kinase [9], MAP kinases [12], and caspases [3,9,12,13]. In addition to its stimulatory effect penta-*O*-galloyl-beta-d-glucose has been shown to counteract apoptosis and cell injury [6,7,10,14].

Similar to apoptosis of nucleated cells, erythrocytes may undergo suicidal death or eryptosis, which is characterized by erythrocyte shrinkage and phosphatidylserine scrambling of the erythrocyte membrane [15]. Triggers of eryptosis include increase of cytosolic Ca^2+^ concentration ([Ca^2+^]_i_), resulting from Ca^2+^ entry through Ca^2+^-permeable cation channels [15,16]. The channels are activated by oxidative stress [15]. Increased [Ca^2+^]_i_ activates Ca^2+^-sensitive K^+^ channels [17,18] leading to cell shrinkage due to K^+^ exit, hyperpolarization and Cl^−^ exit followed by osmotically obliged water [19]. Increased [Ca^2+^]_i_ further triggers phospholipid scrambling of the cell membrane with phosphatidylserine translocation to the erythrocyte surface [20]. The Ca^2+^ sensitivity of cell membrane scrambling is enhanced by ceramide [15]. Further triggers of eryptosis include energy depletion [15], caspase activation [15,21,22], and deranged activity of AMP activated kinase (AMPK) [16], cGMP-dependent protein kinase [23], Janus-activated kinase 3 (JAK3) [24], casein kinase 1α [25,26], p38 kinase [27], as well as sorafenib- [28] and sunitinib- [29] sensitive kinases.

The present study explored the effect of penta-*O*-galloyl-β-d-glucose on cell volume and phosphatidylserine abundance at the erythrocyte surface. As a result, penta-*O*-galloyl-β-d-glucose is a powerful stimulator of eryptosis.

## 2. Results and Discussion

**Figure 1:**
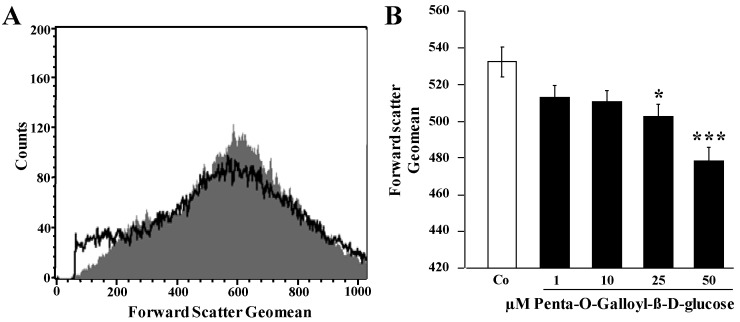
Effect of penta-*O*-galloyl-β-d-glucose (PGG) on erythrocyte forward scatter (**A**) Original histogram of forward scatter of erythrocytes following exposure for 48 h to Ringer solution without (grey) and with (black) the presence of 25 µM penta-*O*-galloyl-β-d-glucose; (**B**) Arithmetic means ± SEM (*n* = 8) of the normalized erythrocyte forward scatter (FSC) following incubation for 48 h to Ringer solution without (white bar) or with (black bars) penta-*O*-galloyl-β-d-glucose (1–50 µM). * (*p* < 0.05), *** (*p* < 0.001) indicates significant difference from the absence of penta-*O*-galloyl-β-d-glucose (ANOVA).

Flow cytometry was employed to explore whether penta-*O*-galloyl-β-d-glucose triggers eryptosis, the suicidal erythrocyte death characterized by cell shrinkage and cell membrane scrambling. Cell volume of human erythrocytes was estimated from forward scatter. As illustrated in Figure 1, a 48-h exposure to penta-*O*-galloyl-β-d-glucose led to a decrease of forward scatter, an effect reaching statistical significance at 25 µM penta-*O*-galloyl-β-d-glucose concentration. Accordingly, penta-*O*-galloyl-β-d-glucose treatment was followed by erythrocyte shrinkage. 

**Figure 2:**
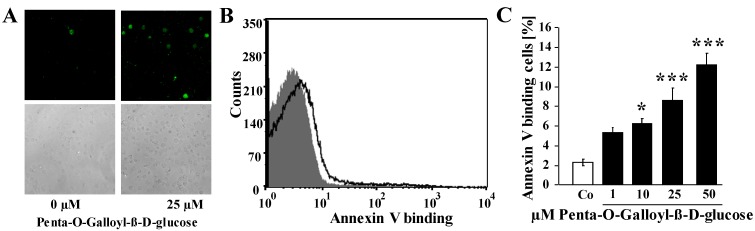
Effect of penta-*O*-galloyl-β-d-glucose (PGG) on phosphatidylserine exposure (**A**) Confocal images of FITC-dependent fluorescence (upper panels) and light microscopy (lower panels) of human erthrocytes following exposure for 48 h to Ringer solution without (left panel) and with (right panel) the presence of 25 µM penta-*O*-galloyl-β-d-glucose; (**B**) Original histogram of annexin V binding of erythrocytes following exposure for 48 h to Ringer solution without (grey) and with (black) the presence of 25 µM penta-*O*-galloyl-β-d-glucose. (**C**) Arithmetic means ± SEM of erythrocyte annexin V binding (*n* = 8) following incubation for 48 h to Ringer solution without (white bar) or with (black bars) the presence of penta-*O*-galloyl-β-d-glucose (1–50 µM). * (*p* < 0.05), *** (*p* < 0.001) indicates significant difference from the absence of penta-*O*-galloyl-β-d-glucose (ANOVA).

In order to elucidate whether penta-*O*-galloyl-β-d-glucose stimulates cell membrane phospholipid scrambling with phosphatidylserine exposure at the erythrocyte surface, phosphatidylserine exposing erythrocytes were identified from annexin V binding determined in flow cytometry. As illustrated in Figure 2, a 48-h exposure to penta-*O*-galloyl-β-d-glucose increased the percentage of annexin V binding erythrocytes, an effect reaching statistical significance at a concentration of 10 µM penta-*O*-galloyl-β-d-glucose. Accordingly, penta-*O*-galloyl-β-d-glucose triggered erythrocyte cell membrane scrambling with phosphatidylserine exposure at the cell surface.

**Figure 3:**
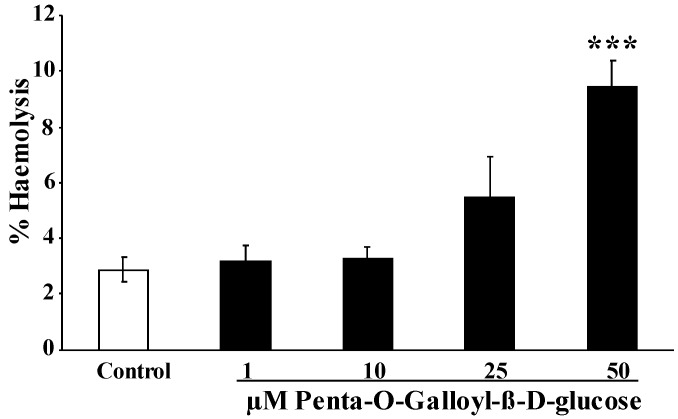
Effect of penta-*O*-galloyl-β-d-glucose (PGG) on hemolysis. Arithmetic means ± SEM of the percentage hemolytic erythrocytes (*n* = 8) following incubation for 48 h to Ringer solution without (white bar) or with (black bars) the presence of penta-*O*-galloyl-β-d-glucose (1–50 µM). *** (*p* < 0.001) indicates significant difference from the absence of penta-*O*-galloyl-β-d-glucose (ANOVA).

To explore whether penta-*O*-galloyl-β-d-glucose exposure triggers hemolysis, the percentage of hemolysed erythrocytes was estimated from hemoglobin concentration in the supernatant. As illustrated in Figure 3, the percentage erythrocytes releasing hemoglobin increased following a 48-h exposure to penta-*O*-galloyl-β-d-glucose concentration, an effect reaching statistical significance at 50 µM penta-*O*-galloyl-β-d-glucose concentration.

Fluo3 fluorescence was employed in order to test, whether penta-*O*-galloyl-β-d-glucose influences the cytosolic Ca^2+^ activity ([Ca^2+^]_i_). To this end, human erythrocytes were loaded with Fluo3-AM and the Fluo3 fluorescence determined by flow cytometry. Prior to determination of Fluo3-fluorescence erythrocytes were incubated in Ringer solution without or with penta-*O*-galloyl-β-d-glucose (1–50 µM). As a result, a 48-h exposure of erythrocytes to penta-*O*-galloyl-β-d-glucose did not significantly modify [Ca^2+^]_i_. The respective arbitrary units (a.u.) approached (*n* = 8 each) 20.29 ± 1.19 a.u., 18.96 ± 1.05 a.u., 17.81 ± 0.93 a.u., 17.61 ± 0.83 a.u., and 22.58 ± 1.37 a.u. after a 48-h exposure to 0, 1, 10, 25, and 50 µM, respectively, of penta-*O*-galloyl-β-d-glucose. 

**Figure 4:**
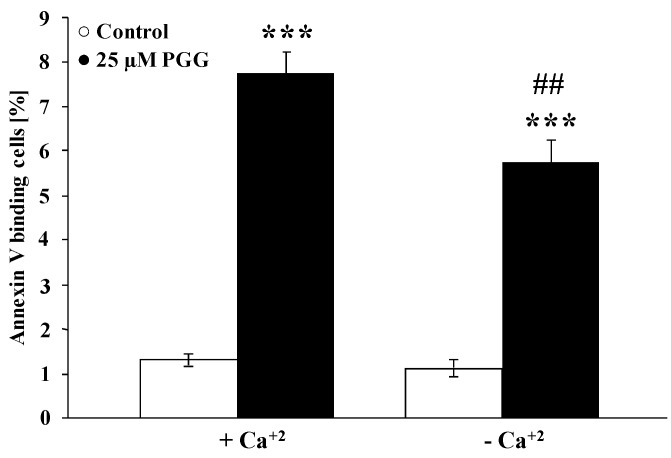
Effect of penta-*O*-galloyl-β-d-glucose (PGG) on phosphatidylserine exposure in the presence and absence of extracellular Ca^2+^. Arithmetic means ± SEM (*n* = 8) of the percentage of annexin V binding erythrocytes after a 48-h treatment with Ringer solution without (white bar) or with (black bars) 25 µM penta-*O*-galloyl-β-d-glucose in the presence (left bars, Plus Calcium) and absence (right bars, Minus Calcium) of calcium. *** (*p* < 0.001) indicates significant difference from the absence of penta-*O*-galloyl-β-d-glucose (ANOVA), ## (*p* < 0.01) indicates significant difference from the respective values in the presence of Ca^2+^ (ANOVA).

Even though penta-*O*-galloyl-β-d-glucose did not significantly modify cytosolic Ca^2+^ activity, the effect of the substance could still be dependent on the presence of Ca^2+^. In order to test this possibility, erythrocytes were exposed to 25 µM penta-*O*-galloyl-β-d-glucose for 48 h in the presence and in the nominal absence of extracellular Ca^2+^. As illustrated in Figure 4, the effect of penta-*O*-galloyl-β-d-glucose on annexin V binding was slightly but significantly blunted in the nominal absence of Ca^2+^. Thus, the effect of penta-*O*-galloyl-β-d-glucose required in part the presence of Ca^2+^. 

**Figure 5:**
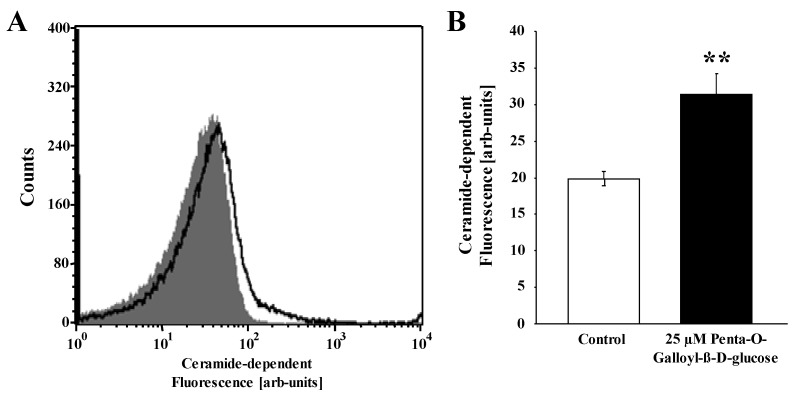
Effect of penta-*O*-galloyl-β-d-glucose (PGG) on ceramide formation. (**A**) Original histogram of anti-ceramide FITC-fluorescence in erythrocytes following exposure for 48 h to Ringer solution without (grey) and with (black) the presence of 25 µM penta-*O*-galloyl-β-d-glucose; (**B**) Arithmetic means ± SEM (*n* = 8) of ceramide abundance after a 48 h incubation in Ringer solution without (white bar) or with (black bars) penta-*O*-galloyl-β-d-glucose (25 µM). ** (*p* < 0.01) indicates significant difference from control (t test).

The sensitivity of cell membrane scrambling to cytosolic Ca^2+^ could be enhanced by ceramide, which is known to trigger eryptosis even at constant [Ca^2+^]i. In order to test, whether ceramide is enhanced by penta-*O*-galloyl-β-d-glucose, ceramide abundance at the cell surface was elucidated utilizing FITC-labeled anti-ceramide antibodies. As shown in Figure 5, penta-*O*-galloyl-β-d-glucose significantly increased ceramide-dependent fluorescence.

The present study discloses a novel xenobiotic triggering suicidal erythrocyte death or eryptosis. Treatment of human erythrocytes with penta-*O*-galloyl-β-d-glucose is followed by erythrocyte shrinkage and erythrocyte cell membrane scrambling, the two hallmarks of eryptosis. The concentrations required (10–50 µM) are similar to those inhibiting hepatocyte apoptosis [14]. 

Penta-*O*-galloyl-β-d-glucose is not paralleled by and thus not due to increase of cytosolic Ca^2+^ activity. Most triggers of eryptosis exert their effect on annexin V binding and cell volume by activation of Ca^2+^ permeable non-selective cation channels in erythrocytes, which involve the transient receptor potential channel TRPC6 [15]. Even though penta-*O*-galloyl-β-d-glucose treatment did not significantly modify cytosolic Ca^2+^ activity, the full effect of penta-*O*-galloyl-β-d-glucose was dependent on the presence of extracellular Ca^2+^. Thus, penta-*O*-galloyl-β-d-glucose appeared to sensitize the erythrocytes to the effects of cytosolic Ca^2+^. A substance known to sensitize erythrocytes to the scrambling effect of enhanced cytosolic Ca^2+^ activity is ceramide [15]. As a matter of fact, penta-*O*-galloyl-β-d-glucose did stimulate ceramide formation.

In nucleated cells, penta-*O*-galloyl-β-d-glucose has been shown to either stimulate [1,2,3,8,9,10,11,12,13] or inhibit apoptosis [6,7,10,14]. A powerful regulator of nuclear cell fate is ceramide, which stimulates death of a wide variety of cells [30]. To the best of our knowledge, penta-*O*-galloyl-β-d-glucose has never been shown before to stimulate the formation of ceramide. Thus, the present observations disclose a mechanism, which could well contribute to triggering of apoptosis. 

The present study may further disclose an effect of penta-*O*-galloyl-β-d-glucose limiting its therapeutic use. Phosphatidylserine exposing erythrocytes adhere to CXCL16/SR-PSO expressed at the luminal cell membrane of endothelial cells [31]. The adherence of the phosphatidylserine exposing erythrocytes to endothelial cells is expected to compromise blood flow and thus to impair microcirculation [31,32,33,34,35,36]. In addition, phosphatidylserine, exposing erythrocytes, may foster blood clotting and, thus, trigger thrombosis [32,37,38]. Phosphatidylserine exposing erythrocytes are further cleared from circulating blood [15]. To the extent that the clearance of erythrocytes exceeds the formation of new erythrocytes, the stimulation of eryptosis may lead to anemia [15]. The therapeutic use of penta-*O*-galloyl-β-d-glucose may, thus, be limited by triggering of eryptosis with subsequent impairment of microcirculation and development of anemia. It should be kept in mind that the sensitivity of erythrocytes to penta-*O*-galloyl-β-d-glucose may be increased by other xenobiotics [15,24,27,39,40,41,42,43,44,45,46,47,48,49,50] and be enhanced in diseases associated with enhanced eryptosis, such as diabetes [15,22,51], renal insufficiency [52], hemolytic uremic syndrome [53], sepsis [54], sickle cell disease [55], malaria [15,56,57], Wilson’s disease [57], iron deficiency [58], phosphate depletion [59], and, presumably, metabolic syndrome [60].

## 3. Experimental Section

### 3.1. Erythrocytes, Solutions, and Chemicals

Leukocyte-depleted erythrocytes were kindly provided by the blood bank of the University of Tübingen. The volunteers (age range 18–68 years) providing blood were tested for HIV, syphilis, and Hepatitis A, B, and C before donation. The study is approved by the ethics committee of the University of Tübingen (184/2003V). Erythrocytes were incubated *in vitro* at a hematocrit of 0.4% in Ringer solution containing (in mM) 125 NaCl, 5 KCl, 1 MgSO_4_, 32 *N*-2-hydroxyethylpiperazine-*N*-2-ethanesulfonic acid (HEPES), 5 glucose, 1 CaCl_2_; pH 7.4 at 37 °C for 48 h. Where indicated, erythrocytes were exposed to penta-*O*-galloyl-β-d-glucose (Sigma, Freiburg, Germany) at the indicated concentrations. In Ca^2+^-free Ringer solution, 1 mM CaCl_2_ was substituted by 1 mM glycol-bis(2-aminoethylether)-*N*,*N*,*N*',*N*'-tetraacetic acid (EGTA). 

### 3.2. Confocal Microscopy and Immunofluorescence

For the visualization of eryptotic erythrocytes, 20 μL erythrocytes were incubated under the respective experimental conditions and then stained with FITC-conjugated Annexin V (1:100 dilution; ImmunoTools, Friesoythe, Germany) in 200 μL Ringer solution containing 5 mM CaCl_2_. Then, the erythrocytes were washed twice and finally resuspended in 100 μL Ringer solution containing 5 mM CaCl_2_. Forty microliters were placed with Prolong Gold antifade reagent (Invitrogen, Darmstadt, Germany) onto a glass slide, covered with a coverslip, and images were subsequently taken on a Zeiss LSM 5 EXCITER confocal laser-scanning microscope (Carl Zeiss MicroImaging, Oberkochen, Germany) with a water immersion Plan-Neofluar 40/1.3 NA DIC.

### 3.3. FACS Analysis of Annexin V Binding and Forward Scatter

After incubation under the respective experimental condition, 50 µL cell suspension was washed in Ringer solution containing 5 mM CaCl_2_ and then stained with Annexin-V-FITC (1:200 dilution; ImmunoTools, Friesoythe, Germany) in this solution at 37 °C for 20 min under protection from light. In the following, the forward scatter (FSC) of the cells was determined, and annexin V fluorescence intensity was measured with an excitation wavelength of 488 nm and an emission wavelength of 530 nm on a FACS Calibur (BD, Heidelberg, Germany).

### 3.4. Measurement of Intracellular Ca^2+^

After incubation erythrocytes were washed in Ringer solution and then loaded with Fluo-3/AM (Biotium, Hayward, CA, USA) in Ringer solution containing 5 mM CaCl_2_ and 5 µM Fluo-3/AM. The cells were incubated at 37 °C for 30 min and washed twice in Ringer solution containing 5 mM CaCl_2_. The Fluo-3/AM-loaded erythrocytes were resuspended in 200 µL Ringer. Then, Ca^2+^-dependent fluorescence intensity was measured with an excitation wavelength of 488 nm and an emission wavelength of 530 nm on a FACS Calibur (BD, Heidelberg, Germany).

### 3.5. Measurement of Hemolysis

For the determination of hemolysis the samples were centrifuged (3 min at 400 g, room temperature) after incubation, and the supernatants were harvested. As a measure of hemolysis, the hemoglobin (Hb) concentration of the supernatant was determined photometrically at 405 nm. The absorption of the supernatant of erythrocytes lysed in distilled water was defined as 100% hemolysis.

### 3.6. Determination of Ceramide Formation

For the determination of ceramide, a monoclonal antibody-based assay was used. After incubation, cells were stained for 1 h at 37 °C with 1 µg/mL anti-ceramide antibody (clone MID 15B4, Alexis, Grünberg, Germany) in PBS containing 0.1% bovine serum albumin (BSA) at a dilution of 1:10. The samples were washed twice with PBS-BSA. Subsequently, the cells were stained for 30 min with polyclonal fluorescein-isothiocyanate (FITC)-conjugated goat anti-mouse IgG and IgM specific antibody (Pharmingen, Hamburg, Germany) diluted 1:50 in PBS-BSA. Unbound secondary antibody was removed by repeated washing with PBS-BSA. The samples were then analyzed at an excitation wavelength of 488 nm and an emission wavelength of 530 nm on a FACS Calibur (BD, Heidelberg, Germany).

### 3.7. Confocal Microscopy and Immunofluorescence

For the visualization of eryptotic erythrocytes, 20 μL erythrocytes were incubated under the respective experimental conditions and then stained with FITC-conjugated Annexin V (1:100 dilution; ImmunoTools) in 200 μL Ringer solution containing 5 mM CaCl_2_. Then, the erythrocytes were washed twice and finally resuspended in 100 μL Ringer solution containing 5 mM CaCl_2_. Forty microliters were placed with Prolong Gold antifade reagent (Invitrogen, Darmstadt, Germany) onto a glass slide, covered with a coverslip, and images were subsequently taken on a Zeiss LSM 5 EXCITER confocal laser-scanning microscope (Carl Zeiss MicroImaging, Oberkochen, Germany) with a water immersion Plan-Neofluar 40/1.3 NA DIC.

### 3.8. Statistics

Data are expressed as arithmetic means ± SEM. As indicated in the figure legends, statistical analysis was made using ANOVA with Tukey’s test as post-test and t test as appropriate. n denotes the number of different erythrocyte specimens studied. As different erythrocyte specimens used in distinct experiments are differently susceptible to triggers of eryptosis, the same erythrocyte specimens have been used for control and experimental conditions.

## 4. Conclusions

Penta-*O*-galloyl-β-d-glucose triggers cell shrinkage and cell membrane scrambling of human erythrocytes and thus eryptosis, the suicidal death of erythrocytes. The effect is in part dependent on the presence of extracellular Ca^2+^ and is at least in part due to stimulation of ceramide formation. 
